# Upper extremity versus lower extremity for secondary access during transcatheter aortic valve implantation: rationale and design of the randomised TAVI XS trial

**DOI:** 10.1007/s12471-024-01869-5

**Published:** 2024-04-23

**Authors:** Maxim J. P. Rooijakkers, Geert A. A. Versteeg, Kimberley I. Hemelrijk, Hugo M. Aarts, Daniël C. Overduin, Dirk-Jan van Ginkel, Pieter J. Vlaar, Marleen H. van Wely, Lokien X. van Nunen, Robert Jan van Geuns, Leen A. F. M. van Garsse, Guillaume S. C. Geuzebroek, Michel W. A. Verkroost, Laura Rodwell, Robin H. Heijmen, Pim A. L. Tonino, Jurrien M. ten Berg, Ronak Delewi, Niels van Royen

**Affiliations:** 1 Department of Cardiology, Radboud University Medical Centre, Nijmegen, The Netherlands; 2 Department of Cardiology, Amsterdam University Medical Centre, Amsterdam, The Netherlands; 3 Department of Cardiology, St. Antonius Hospital, Nieuwegein, The Netherlands; 4 Department of Cardiology, Catharina Hospital, Eindhoven, The Netherlands; 5 Department of Cardiothoracic Surgery, Radboud University Medical Centre, Nijmegen, The Netherlands; 6 Department of Health Sciences, Section Biostatistics, Radboud Institute for Health Sciences, Nijmegen, The Netherlands; 7 Cardiovascular Research Institute Maastricht, Maastricht, The Netherlands

**Keywords:** Aortic stenosis, Bleeding complications, Pacemaker, Transcatheter aortic valve implantation, Valve Academic Research Consortium 3, Complications

## Abstract

**Background:**

During transcatheter aortic valve implantation (TAVI), secondary access is required for angiographic guidance and temporary pacing. The most commonly used secondary access sites are the femoral artery (angiographic guidance) and the femoral vein (temporary pacing). An upper extremity approach using the radial artery and an upper arm vein instead of the lower extremity approach using the femoral artery and femoral vein may reduce clinically relevant secondary access site-related bleeding complications, but robust evidence is lacking.

**Trial design:**

The TAVI XS trial is a multicentre, randomised, open-label clinical trial with blinded evaluation of endpoints. A total of 238 patients undergoing transfemoral TAVI will be included. The primary endpoint is the incidence of clinically relevant bleeding (i.e. Bleeding Academic Research Consortium (BARC) type 2, 3 or 5 bleeding) of the randomised secondary access site (either diagnostic or pacemaker access, or both) within 30 days after TAVI. Secondary endpoints include time to mobilisation after TAVI, duration of hospitalisation, any BARC type 2, 3 or 5 bleeding, and early safety at 30 days according to Valve Academic Research Consortium‑3 criteria.

**Conclusion:**

The TAVI XS trial is the first randomised trial comparing an upper extremity approach to a lower extremity approach with regard to clinically relevant secondary access site-related bleeding complications. The results of this trial will provide important insights into the safety and efficacy of an upper extremity approach in patients undergoing transfemoral TAVI.

## Introduction

Transcatheter aortic valve implantation (TAVI) is an established treatment for patients with severe aortic stenosis (AS) across the entire spectrum of surgical risk [1, 2]. Increased operator experience, reduction in sheath size and improved percutaneous closure have resulted in decreased bleeding complications of the primary access site [3].

Little attention has been paid to secondary access. Additional arterial access is required for angiographic guidance, haemodynamic monitoring and management of complications at the primary TAVI access site. Venous access is required if a temporary pacing lead is inserted. Although most access site complications during TAVI are related to primary access, up to one-fourth of bleeding and vascular complications are related to transfemoral secondary access, negatively impacting patient outcome [4, 5].

Data on alternative diagnostic access sites are scarce. Currently available retrospective data show a reduction in bleeding and vascular complications associated with using the radial artery for secondary access. However, these data are derived from small non-randomised studies with considerable methodological limitations [4–6].

Rapid ventricular pacing is used when implanting balloon-expandable devices and during pre- and post-dilation. In addition, pacing can assist in proper valve positioning and deployment, and provides a direct back-up in case of conduction disturbances. During the first 24 h post-TAVI, the temporary pacing lead is frequently left in place to overcome conduction disturbances, depending on peri-procedural electrocardiographic characteristics [7]. In current practice, the femoral vein is the most frequently used temporary pacemaker access. However, due to its anatomical position and large vessel diameter, access site haematomas are common, occurring in approximately 4% of patients [8]. An upper arm vein can serve as alternative pacemaker access, but data are lacking in patients undergoing TAVI.

Given the complication risk associated with the primary access, it is necessary to search for strategies to minimise additional complication risks. Using the upper extremity instead of the lower extremity for diagnostic and pacemaker access potentially reduces this risk. Therefore, we have designed the randomised TAVI XS trial, aiming to assess the safety and efficacy of an upper extremity approach versus a lower extremity approach for secondary access site-related bleeding in patients undergoing transfemoral TAVI.

## Methods

### Study design

The TAVI XS trial is an investigator-initiated, multicentre, randomised trial comparing an upper extremity approach to a lower extremity approach regarding secondary access during TAVI. Participating centres are Radboud University Medical Centre (Nijmegen, The Netherlands), Amsterdam University Medical Centre (Amsterdam, The Netherlands), Catharina Hospital (Eindhoven, The Netherlands) and St. Antonius Hospital (Nieuwegein, The Netherlands). This trial has been approved by the Medical Research Ethics Committee Oost-Nederland and by the institutional review board of each participating site. Written informed consent is obtained from all patients prior to enrolment. The trial was designed in accordance with the Declaration of Helsinki. All data are collected in Castor (Castor EDC, Amsterdam, The Netherlands). Evaluation of serious adverse events (SAEs) is performed by an independent data safety monitoring board (DSMB), which convenes when 50% of the patients have reached the 30-day follow-up. A clinical event committee will review and adjudicate all endpoint-related AEs. Monitoring of the trial is executed by the Radboudumc Technology Centre Clinical Studies. The TAVI XS trial has been registered in the ClinicalTrials.gov database (NCT05672823).

### Inclusion

**Fig. 1:**
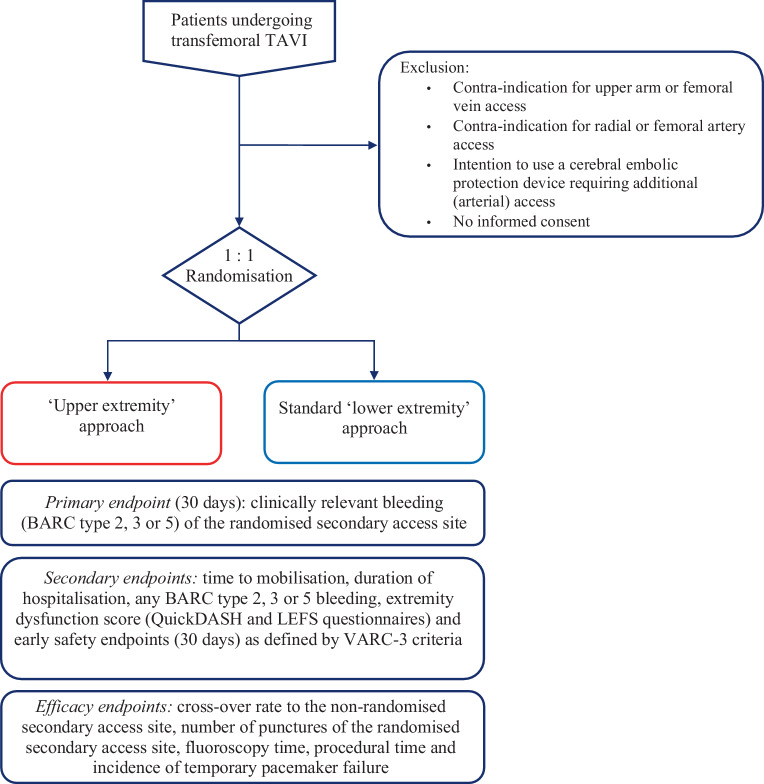
Flowchart of the TAVI XS trial. *BARC* Bleeding Academic Research Consortium, *QuickDASH* Quick Disabilities of Arm, Shoulder and Hand, *LEFS* Lower Extremity Functional Scale, *TAVI* transcatheter aortic valve implantation, *VARC* Valve Academic Research Consortium

All patients ≥ 18 years scheduled for transfemoral TAVI are screened for inclusion. Patients with a contra-indication for upper arm or femoral vein access, contra-indication for radial or femoral artery access, or patients in whom there is an intent to use a cerebral embolic protection device requiring additional (arterial) access are excluded (Fig. 1).

### Randomisation

Eligible patients are randomly assigned to receive one of the two study treatments in a 1:1 ratio. Randomisation is performed within Castor, with variable block sizes of 2 and 4, and stratification according to study site and use of dual antiplatelet therapy and/or oral anticoagulants at baseline.

### Study endpoints

The primary endpoint is defined as clinically relevant bleeding (i.e. Bleeding Academic Research Consortium (BARC) type 2, 3 or 5) [9] of the randomised secondary access site (either diagnostic or pacemaker access, or both) within 30 days after TAVI. If clinically relevant bleeding occurs at both the diagnostic and pacemaker access site, the highest classification of the two BARC bleedings is scored. Secondary endpoints include time to mobilisation, duration of hospitalisation, any BARC type 2, 3 or 5 bleeding, extremity dysfunction score (assessed with the Quick Disabilities of the Arm, Shoulder and Hand [10] and Lower Extremity Functional Scale [11] questionnaires at baseline and 1‑month follow-up) and early safety (30 days) as defined by Valve Academic Research Consortium‑3 criteria [12].

Efficacy endpoints include cross-over rate to the non-randomised secondary access site (defined as conversion from upper to lower extremity or vice versa, which applies to either diagnostic or pacemaker access, or both; conversion to the contralateral upper or lower extremity is not considered a cross-over, but these data will be collected), number of punctures of the randomised secondary access site, fluoroscopy time, procedural time and incidence of temporary pacemaker failure.

### TAVI procedure

**Fig. 2:**
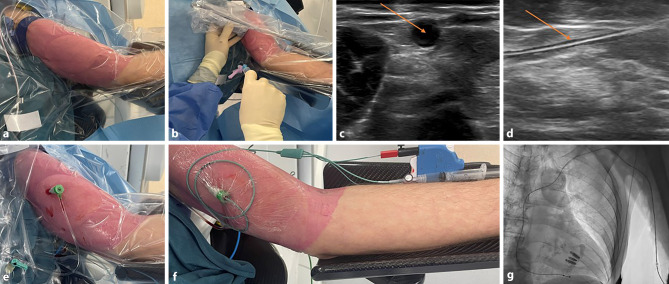
**a**–**g** Procedural steps in temporary pacing lead placement in an upper arm vein. **a** Positioning of the upper arm. **b**, **c** Ultrasound-guided visualisation of the upper arm vein. *Arrow* indicates the upper arm vein. **d** Guidewire entering the upper arm vein. *Arrow* indicates the guidewire. **e** 6F sheath inserted in the upper arm vein. **f** Fixation of 5F temporary pacing lead inserted through a 6F sheath in the upper arm vein. **g** Angiographic visualisation of the temporary pacing lead trajectory from a left upper arm vein to right ventricular apex

TAVI is performed according to the local protocol of each participating site. In patients randomised to the upper extremity approach, the radial artery is used for diagnostic access. An upper arm vein is used for temporary pacemaker access (Fig. 2); alternatively, pacing over the left ventricular (LV) stiff wire can be used (left to the operator’s discretion). In patients randomised to the lower extremity approach, the contralateral femoral artery (femoral artery not used for TAVI access) is used for diagnostic access. The femoral vein is used for temporary pacemaker access; alternatively, pacing over the wire can be used (operator’s discretion). The decision regarding venous access or pacing over the wire is made prior to randomisation. When pacing over the wire is performed, venous access will not be routinely obtained. All sites are instructed to use ultrasound (US) guidance to obtain femoral artery, femoral vein and upper arm vein access. The radial artery could also be punctured using US guidance, but this is left to the discretion of the operator.

Upper arm venous access is obtained by puncturing a robust vein without relevant anatomical structures in its direct proximity. A tourniquet is used to improve visualisation of the vein. A more detailed instruction of this approach has been described previously [13].

### Follow-up

Follow-up is performed at discharge and at 30 days post-TAVI, either on-site or by phone call. All SAEs are documented from inclusion to 30-day follow-up and are assessed by an independent DSMB, composed of two experienced cardiologists and an epidemiologist/statistician.

### Sample size calculation and statistics

We anticipate an incidence of the primary endpoint of 2% in the upper extremity group and 12% in the lower extremity group, based on previous studies investigating the bleeding rate of secondary access sites [8, 14]. Based on a superiority design with a type I error of 5% and a power of 80%, a total of 216 patients will be needed. Assuming a 10% loss to follow-up rate, a total of 238 patients will be needed.

The primary analysis will take place after the last patient follow-up, using an intention-to-treat approach. Categorical data will be presented as frequencies and proportions. Continuous data will be presented as mean and standard deviation or as median and interquartile range, as appropriate. The chi-square test will be used for the primary endpoint. Superiority will be tested to evaluate the safety of an upper extremity over a lower extremity approach. Superiority is proven if the two-sided *p*-value is < 0.05. Besides the intention-to-treat analysis, a secondary, separate ‘as treated’ analysis will be performed for the primary endpoint.

## Discussion

Access site-related bleeding events are independently associated with an increased risk of mortality after TAVI [15]. In addition, peri-procedural bleeding complications of the primary access site are associated with a more than doubled increase in in-hospital mortality, longer duration of hospitalisation and higher healthcare costs [16]. Over the past years, technical refinements, increased operator experience and the widespread adoption of transfemoral access as the default TAVI approach have resulted in a reduction in peri-procedural bleeding complications predominantly related to the primary access site [3, 17]. In contrast, secondary (both diagnostic and temporary pacemaker) access site-related complications have been substantially less studied, despite being responsible for up to a quarter of vascular and bleeding complications during TAVI, adversely affecting patient outcome [4, 18]. The TAVI XS trial is designed to address this knowledge gap, aiming to assess the safety and efficacy of an upper extremity approach instead of a lower extremity approach in terms of clinically relevant secondary access site-related bleeding complications in patients undergoing transfemoral TAVI.

Considering the previously mentioned non-negligible incidence of bleeding complications related to femoral secondary access, it seems sensible to search for alternative approaches to ensure angiographic guidance during TAVI. A wealth of data exists on the optimal approach for patients undergoing coronary angiography with or without percutaneous coronary intervention, supporting the use of the radial artery over the femoral artery in patients undergoing myocardial revascularisation [19]. In the TAVI population, however, robust data on the optimal diagnostic access are lacking. Current evidence is limited to small non-randomised retrospective and mainly single-centre studies. In a study by Allende et al., it was shown that transradial secondary access was associated with a significant reduction in major and/or life-threatening bleeding and major vascular complications compared with transfemoral secondary access [5]. The same results were shown by Fernandez-Lopez et al., who described a significant decrease in bleeding and vascular complications associated with a transradial approach, albeit with an increase in fluoroscopy time [6]. In a propensity-matched multicentre analysis by Junquera et al., transradial secondary access was associated with a significant reduction in bleeding and vascular complications, as well as improved 30-day outcomes [4]. The above-mentioned studies were included together with three other studies in a meta-analysis by Das et al. in 2022, showing consistent results regarding the reduction in bleeding (odds ratio (OR) 0.46, 95% confidence interval (CI) 0.36–0.59) and vascular (OR 0.58, 95% CI 0.43–0.77) complications in the transradial cohort when compared to the transfemoral cohort [20].

Compared to the diagnostic access, even less is known about the occurrence of access site-related bleeding complications of the temporary pacemaker access site. This access is needed for rapid ventricular pacing, which is an essential part of the TAVI procedure to ensure cardiac standstill during valve positioning and deployment, as well as during pre- and post-dilation. This is typically achieved using a transvenous temporary pacing lead positioned in the apex of the right ventricle (right ventricular (RV) pacing). In patients without pre-procedural right bundle branch block and without post-procedural electrocardiographic changes, the temporary pacing lead is removed at the end of the TAVI procedure. In all other cases, the temporary pacing lead is usually left in place for at least 24 h post-TAVI to provide direct back-up in case of conduction disturbances [7]. Conventionally, the femoral vein is used for temporary pacemaker access, whereas in patients undergoing TAVI under general anaesthesia the jugular vein is frequently used. With the widespread adoption of the minimalist TAVI approach, the use of general anaesthesia is minimised, and hence the use of the jugular vein as temporary pacemaker access is markedly reduced. Moreover, jugular vein access is associated with a higher number of access site-related bleeding complications as compared with femoral vein access [21]. We previously showed that upper arm vein access is safe and feasible, and that its use is associated with fewer bleeding complications as compared with non-upper arm vein access [13]. However, it should be noted that this was an observational study, in which no randomised comparison was made between the upper arm and femoral approach. Besides the potentially higher bleeding risk as compared with an upper arm vein, an important disadvantage of using the femoral vein for temporary pacemaker access is the inability of a patient to mobilise while the temporary pacing lead is still in place, in order to prevent dislocation of the lead. Prolonged immobilisation is a well-established risk factor for post-operative delirium and urinary tract infections [22, 23], which in turn may lead to a prolonged hospitalisation with inherently increased healthcare costs.

In attempts to further refine the TAVI procedure, pacing over the LV stiff wire is emerging as an alternative to RV pacing. This technique does not require venous sheath placement for insertion of a temporary pacing lead; instead, rapid ventricular pacing is performed via the LV guidewire [24]. Hence, the number of access sites required during TAVI is reduced, thereby potentially mitigating the risk of vascular and bleeding complications. In the EASY TAVI trial, it was shown that LV pacing via the valve delivery guidewire was associated with a significant reduction in procedural duration, fluoroscopy time and costs, with similar efficacy and safety compared with RV pacing [25]. Similar results regarding procedural duration were shown in a study by Hokken et al. No issues with ventricular capture were observed in the LV pacing cohort [26]. In the TAVI XS trial, the decision to use RV pacing or LV pacing is left to the discretion of the operator.

An alternative attempt to reduce bleeding complications has been described by Khubber et al., who investigated the possibility of a unilateral access transfemoral TAVI approach [27]. This approach could be beneficial for primary access site management, as no femoral cross-over is required. Management of the primary access site using the radial artery can be challenging and is not always possible. In these cases, a femoral cross-over strategy requires a third arterial access, which should be considered when assessing the potential benefit of transradial secondary access.

In conclusion, the TAVI XS trial is the first prospective, investigator-initiated, multicentre randomised trial comparing an upper extremity approach to a lower extremity approach with regard to clinically relevant secondary access site-related bleeding complications. The results of this trial will provide important insights into the safety and efficacy of an upper extremity approach in patients undergoing transfemoral TAVI. If this trial can show that the use of the upper extremity is associated with fewer clinically relevant secondary access site-related bleeding complications, it can be an important step in further reducing the invasiveness of TAVI.
